# Dissection de l´aorte abdominale révélatrice de la maladie de Takayasu: à propos d´un cas en Guinée

**DOI:** 10.11604/pamj.2020.37.34.21441

**Published:** 2020-09-08

**Authors:** Barry Ibrahima Sory, Balde El Hadj Yaya, Camara Abdoulaye, Samoura Aly, Koivogui Diarra, Koivogui Kokoulo, Soumaoro Morlaye, Sylla Djibril, Bah Mamadou Bassirou, Beavogui Mariame, Balde Mamadou Dadhi, Conde Mamady

**Affiliations:** 1 Service de Cardiologie de l´Hôpital National Ignace Deen, Centre Hospitalier Universitaire de Conakry, Conakry, République de Guinée,; 2 Service des Urgences Médico-Chirurgicales de l'Hôpital National Donka, Centre Hospitalier Universitaire de Conakry, Conakry, République de Guinée

**Keywords:** Maladie Takayasu, dissection, aorte abdominale, Takayasu´s disease, dissection, aorta, abdominal

## Abstract

La maladie de Takayasu (MT) est une artériopathie inflammatoire chronique touchant l´aorte, ses principales branches et les artères pulmonaires. Son appellation tient de l´ophtalmologiste japonais Mikito Takayasu qui publia en 1908 la première description de la maladie. Il s´agissait d´un patient de 78ans admis pour douleur abdominale, douleur du membre inférieur droit à la marche, insomnie. Evoluant depuis 1an sans antécédent de maladie cardio-vasculaire connu. A l´examen physique: le rythme cardiaque régulier à 87 battements par minute sans bruits pathologiques surajoutés avec une absence de pouls pédieux droit, tension artérielle à 120/78 mmhg, poumons libres, abdomen souple avec une masse battante dans la fosse iliaque droite dont l´auscultation met en évidence un souffle continu. Le reste de l´examen est sans particularité. L´angioscanner abdominal confirmait un aspect de dissection aortique étendue sur l´ensemble de l´aorte abdominale avec opacification synchrone des deux chenaux, un anévrisme thrombosé des artères iliaques primitives mesurant 48mm x100mm à droite et 38mm x 90mm à gauche, absence de fissuration visible. Nous rapportons le cas d´une dissection de l´aorte abdominale associée à un anévrisme thrombosé des artères iliaques primitives révélant une maladie de Takayashu au service de cardiologie de l´hôpital national Ignace Deen. La fréquence de la dissection de l´aorte abdominale au cours de la maladie de Takayasu est rare. Elle est plus souvent diagnostiquée dans sa phase occlusive. Le pronostic dépend des complications évolutives.

## Abstract (en)

Takayasu´s disease (TD) is a chronic inflammatory arteriopathy affecting the aorta, its main branches and the pulmonary arteries. It was first described in 1908 by the Japanese ophthalmologist Mikito Takayasu. The study involved a 78-year-old patient with no known history of cardiovascular disease, admitted with abdominal pain, pain in the right lower limb when walking, insomnia. These symptoms had progressed over 1 year. Physical examination showed normal heart rate of 87 beats per minute without pathological noises, with absence of right pedal pulse, blood pressure 120/78 mmhg, free lungs, flexible abdomen, and a beating mass in the right iliac fossa whose auscultation showed continuous murmur. The remainder of the physical examination was normal. Abdominal CT angiography confirmed extensive aortic dissection at the abdominal aorta with synchronous opacification of both channels, thrombotic aneurysm of the primitive iliac arteries measuring 48mmx100mm on the right and 38mm x 90mm on the left, with no visible fissurization. We report a case abdominal aorta dissection associated with thrombus formation in the primary iliac arteries revealing Takayashu´s disease treated in the Department of Cardiology at the Ignace Deen National Hospital. The rate of abdominal aorta dissection in patients with Takayasu´s disease is rare. It is most often diagnosed in the occlusive phase. Prognosis depends on possible complications.

## Introduction

La maladie de Takayasu (MT) est un artériopathie inflammatoire chronique touchant l´aorte, ses principales branches et les artères pulmonaires. Son appellation tient de l´ophtalmologiste japonais Mikito Takayasu qui publia en 1908 la première description de la maladie [1]. Elle occasionne ainsi des lésions à type de sténoses, d´occlusions ou d´anévrismes dans les territoires atteints [2,3]. Le grand polymorphisme clinique de cette maladie fait que le diagnostic est rarement posé à sa phase systémique ou « préocclusive » et c´est souvent des signes ischémiques qui la révèlent à sa phase « occlusive » [4].

## Patient et observation

**Figure 1:**
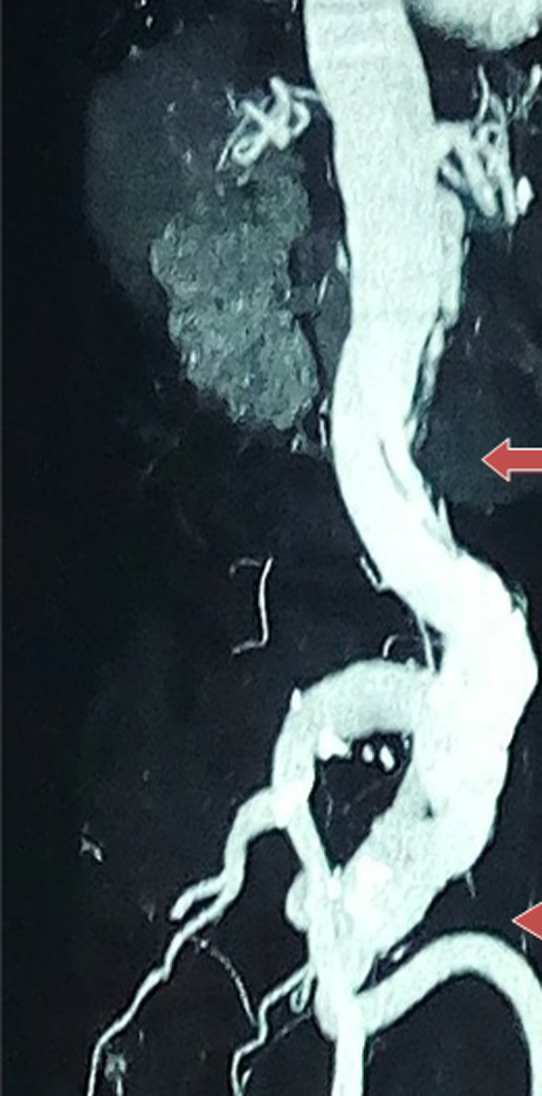
angioscanner abdominal montrant un aspect de dissection aortique étendue sur l´ensemble de l´aorte abdominale avec opacification synchrone des deux chenaux, un anévrisme thrombosé des artères iliaques primitives

Il s´agissait d´un patient de 78 ans admis pour douleur abdominale, douleur du membre inférieur droit à la marche, insomnie. Evoluant depuis 1an sans antécédent de maladie cardio-vasculaire connu. A l´examen physique: le rythme cardiaque régulier à 87 battements par minute sans bruits pathologiques surajoutés avec une absence de pouls pédieux droit, tension artérielle à 120/78mmhg, poumons libres, abdomen souple avec une masse battante dans la fosse iliaque droite dont l´auscultation met en évidence un souffle continu. Le reste de l´examen est sans particularité. Température à 37,5°C. L´électrocardiogramme inscrivait un bloc de branche gauche complet. L´examen biologique montrait une vitesse de sédimentation (VS) accélérée à 55mm première heure et 68mm deuxième heure, la protéine C-réactive (CRP) élevée à 98mg, Widal négatif, HIV négatif, l´intra dermoréaction (IDR) à la tuberculine négative. L´échographie abdomino-pelvienne concluait à un anévrisme partiellement thrombosé des artères iliaques externes mesurant 52mm à droite et 44mm à gauche, à un flap endoluminal de l´aorte abdominale. L´angio-scanner abdominal confirmait un aspect de dissection aortique étendue sur l´ensemble de l´aorte abdominale avec opacification synchrone des deux chenaux, un anévrisme thrombosé des artères iliaques primitives mesurant 48mm x 100mm à droite et 38mm x 90mm à gauche, absence de fissuration visible (Figure 1). Au terme de l´examen clinique, paraclinique et vu les critères de l´American College of Rheumatology, le diagnostic de la maladie de Takayasua été retenu.

## Discussion

Nous rapportons le cas d´une dissection de l´aorte abdominale associée à un anévrisme thrombosé des artères iliaques primitives révélant une maladie de Takayashu au service de cardiologie de l´hôpital national Ignace Deen. La maladie de Takayasu est une artérite inflammatoire chronique d'origine inconnue qui affecte les vaisseaux de gros calibre principalement l'aorte et ses branches principales [5]. La prévalence de la maladie de Takayasu est plus importante au Japon, en Asie du Sud-Est, au Mexique, en Amérique latine et en Afrique. L´incidence annuelle est estimée entre 2 à 3 cas par million d´habitants [5]. En Guinée, il n´ya pas de donnée publiée sur la maladie de Takayashu. La MT touche préférentiellement la femme dans sa troisième décennie, mais l´âge de début est très variable [6,7]. Notre sujet était du genre masculin presque octogénaire en opposition avec la littérature.

Sur le plan clinique, la diversité et le manque de spécificité des symptômes de la phase précoce, dite pré-occlusive expliquant le retard diagnostique [8]. Le diagnostic de la maladie ne se fait généralement qu´à la phase occlusive où les symptômes ischémiques deviennent francs avec des délais qui peuvent atteindre une dizaine d´année [9]. La phase occlusive, qui peut être d'emblée la phase unique de cette maladie, sous forme de manifestation vasculaire (ischémique, sténosante, anévrismale, disséquant) avec ou sans syndrome inflammatoire [10]. Notre patient présentait une dissection aortique étendue sur l´ensemble de l´aorte abdominale, un anévrisme thrombosé des artères iliaques primitives. La claudication des membres à l´effort, qui se voit plus souvent aux membres supérieurs qu´inférieurs était le signe révélateur de la maladie chez la majorité des patients de notre étude (81,5 %), associée pour certains à des signes neurosensoriels d´HTA (37%) et/ou des signes généraux (29,6%) [4,9,11]. Notre patient présentait une claudication des membres inférieurs et une douleur abdominale. La claudication intermittente des membres inférieurs peut révéler la coexistence de sténoses et de dilatations ou anévrisme de l´aorte thoracique ou l´aorte abdominale, très évocateurs de la maladie, surtout lorsque la paroi vasculaire est épaissie [12]. La cause de la maladie demeure inconnue et plusieurs hypothèses sont évoquées [12].

Le diagnostic de maladie de Takayasu repose sur un faisceau d´arguments cliniques et d´imagerie: le sexe féminin, l´âge ≤ 40 ans, le syndrome inflammatoire biologique, l´absence de facteurs de risque majeurs d´athérosclérose et la localisation à l´aorte et ses branches. Les critères proposés par l´American College of Rheumatology (ACR) aident à classer cette artérite [13]. Notre patient présentait 3 des 6 critères de l´ ACR pour poser le diagnostic de la MT en plus du syndrome inflammatoire qui sont: claudication des extrémités, Souffle auscultatoire sur une artère sous-clavière ou sur l´aorte abdominale et Anomalies artériographiques non liées à l´athérosclérose. La rareté de la MT explique le manque des essais contrôlés du traitement médical. La chirurgie vasculaire de la dissection aortique est complexe et grevée d'une lourde mortalité ou de séquelles paraplégiques [14]. Ce genre d'intervention doit être effectué par une équipe très expérimentée.

## Conclusion

La fréquence de la dissection de l´aorte abdominale au cours de la maladie de Takayasu est rare. Elle est plus souvent diagnostiquée dans sa phase occlusive. Le pronostic dépend des complications évolutives.

## References

[1] Chun YS, Park SJ, Park IK, Chung H, Lee J (2001). The clinical and ocular manifestations of Takayasuarteritis. Retina.

[2] Hachulla E, Bérégi JP (2001). Diagnostic des aortites. J Mal Vasc.

[3] David Launay, Eric Hachulla (2004). Les aortites inflammatoires. Presse Med.

[4] Ghannouchi Jaafouraa N, Khalifaa M, Rezguia A, Alaouaa E, Ben Jaziaa A, Braham A (2010). La maladie de Takayasu dans la région centre de la Tunisie: a propos de 27 cas. Journal des Maladies Vasculaires.

[5] Abdelmajid Bouzerda, Ali khatouri (2016). Manifestations cardiaques de la maladie de Takayasu: à propos d´une observation et revue de la literature. Pan African Medical Journal.

[6] Lupi-Herrera E, Sanchez-Torres G, Marcushamer J, Mispireta J, Horwitz S, Vela JE (1977). Takayasu´sarteritis: clinical study of 107 cases. Am Heart J.

[7] Hotchi M (1992). Pathological studies on Takayasu´sarteritis. Heart Vessels Suppl.

[8] Piette AM, Blétry O (1997). Maladie de Takayasu In: Maladies et syndromes systémiques. 4e ed Paris: Med Sc Flammarion.

[9] Sato EI, Hatta FS, Levy-Neto M, Fernandes S (1998). Demographic, clinical, and angiographic data of patients with Takayasuarteritis in Brazil. Int J Cardiol.

[10] Arnaud J, Haroche J, Piette C, Amoura Z (2010). L´artérite de Takayasu: mise au point à propos d'une série momocentrique de 82 patients. Rev Med Interne.

[11] El Asri A, Tazi-Mezalek Z, Aouni M, Adnaoui M, Mohattane A, Bensaid Y (2002). La maladie de Takayasu au Maroc: à propos de 47 observations. Rev Med Interne.

[12] Mirault T, Messas E (2016). La maladie de Takayasu. Rev Med Interne.

[13] Arend WP, Michel BA, Bloch DA, Hunder GG, Calabrese LH, Edworthy SM (1990). The American College of Rheumatology 1990 criteria for the classification of Takayasuarteritis. Arthritis Rheum.

[14] Abdelaziz Zaghdoudi, Monika Bukta, Mohamed Ali Mongalgi, Kais Malouche, Sonia Malouche (2014). Dissection de l´aorte thoracique descendante et de l´aorte abdominale dans la maladie de Takayasu: à propos d´un cas. Pan AfricanMedical Journal.

